# How reliable is BMI? Bioimpedance analysis of body composition in underweight, normal weight, overweight, and obese women

**DOI:** 10.1007/s11845-020-02403-3

**Published:** 2020-10-21

**Authors:** Agata Lebiedowska, Magdalena Hartman-Petrycka, Barbara Błońska-Fajfrowska

**Affiliations:** Department of Basic Biomedical Science, Faculty of Pharmaceutical Sciences in Sosnowiec, Medical University of Silesia, Katowice, Poland, Kasztanowa 3, 41-200 Sosnowiec, Poland

**Keywords:** Bioelectrical impedance analysis (BIA), BMI, Body composition

## Abstract

**Background:**

The human body consists of water, proteins, lipids, carbohydrates, and minerals that build cells, tissues, and organs. In healthy people, the content of these molecules remains nearly constant. The body mass index (BMI) is commonly used to classify abnormal body composition among adults. According to the WHO, a high BMI is a major risk factor for many diseases. Bioelectrical impedance analysis is a commonly used method for assessing body composition in clinical practice and medical research.

**Aims:**

The aim of this study was to identify the advantages and disadvantages of using BMI in diagnosis of underweight, overweight, and obesity, by comparing the value of the index with the values of body composition analysis parameters.

**Methods:**

A total of 267 healthy women 18 to 35 years of age participated in this study. Bioelectrical impedance analysis was performed on all participants at the beginning of the experiment with an InBodyS10 device.

**Results:**

In the BMI categories of overweight and obese, only women with excessive BFM were measured with BIA. The BMI category of normal body weight, apart from women with normal body composition, includes people with both deficient and excess body components, e.g., body fat or lean body mass. The BMI category of underweight includes women with different body compositions as well as people with excessive fat content.

**Conclusions:**

The BMI is useful to provide a warning of excessive fat content in overweight and obese women, whereas among normal weight and underweight women, it may mask various types of body composition defects.

**Electronic supplementary material:**

The online version of this article (10.1007/s11845-020-02403-3) contains supplementary material, which is available to authorized users.

## Introduction

Currently, there is a growing dissonance between the development of civilization and human health. Consequently, hurried eating, consuming irregular and inadequately balanced meals, performing sedentary work, transport mainly by car, and a lack of time and discipline to perform physical exercise all contribute to the development of diseases such as obesity, diabetes, or atherosclerosis [1, 2]. The World Health Organization (WHO) has warned that over the past 30 years, the number of obese people worldwide has doubled. In 2014, nearly 2 billion people over the age of 18 had excessive weight, 600 million of whom were obese, that is, 39% of the adult population was overweight, and 13% was obese. This problem is concerning for the entire world, with the exception of only African countries and some South Asian countries. In the twenty-first century, the obesity epidemic is killing more people than hunger [3, 4]. To control human body weight, several measurement methods have been developed, including simple anthropometric methods and more advanced devices [5]. In this work, the most commonly used anthropometric index, the body mass index (BMI), was compared with the results of bioelectrical impedance analysis (BIA), thus enabling body composition analysis of parameters including fat mass (FM) content. The BMI is a simple indicator commonly used to classify abnormal body composition in adults. It is defined as the mass of a person in kilograms divided by the square of the height in meters (kg/m^2^). A BMI greater than or equal to 25 is considered overweight, and a BMI greater than or equal to 30 is considered obese. According to the WHO, a high BMI is a major risk factor for diseases such as cardiovascular disease (including heart disease and stroke), diabetes, musculoskeletal disorders (especially osteoarthritis), and some cancers (including those of the endometrium, breast, ovaries, prostate, liver, gallbladder, kidney, and large intestine) [4].

BIA is a commonly used method for assessing body composition in clinical practice and scientific research. The principle of the method is based on the difference in the conduction of electric current through water and fat in the human body. Extracellular and intracellular fluids of living organisms are electric conductors, whereas cell membranes are low-reactive electric capacitors. Low-frequency current (approximately 1 kHz) flows primarily through extracellular fluids, whereas a current of approximately 500–800 kHz flows through both extracellular and intracellular fluids [3]. Measurement of electrical impedance along with individual information such as height, weight, and sex enables the estimation of body composition parameters in the human body.

The human body consists of water, proteins, lipids, carbohydrates, and minerals, which together make up the body weight. These components build cells, tissues, and organs. Proteins are major components of muscles, fats are major components of lipids, and minerals are major components of bone tissues. In healthy individuals, the content of these ingredients remains nearly constant. Total body water (TBW) is the sum of extracellular body water (ECW) and intracellular body water, which is mainly found in the muscles and internal organs. The basic two-component model assumes that the human body is composed of body fat mass (BFM) and fat-free mass (FFM). Within the FFM, bone minerals and so-called soft lean mass (SLM) are distinguished. SLM consists of body cell mass (BCM) and extracellular water, whereas the BCM is made of proteins and intracellular water [6, 7]. The BFM consists of subcutaneous fat located directly under the skin, which makes up most of the lower body fat (thighs and buttocks) and the visceral fat area (VFA) in the abdomen. The healthful range of percentage body fat in women is 18 to 28%. Men have a lower FM than women and a higher content of fat-free components, such as skeletal muscle mass (SMM) and bone mass. Studies have indicated no differences in VFA between men and women [8].

BIA measurement is simple and does not require special operator skills or substantial patient involvement; thus, it is a fast, safe, and easy method [5].

The aim of this study was to identify the advantages and disadvantages of using BMI in diagnosing underweight, overweight, and obesity by comparing the value of this indicator with the values of body composition analysis parameters.

## Materials and methods

A total of 267 healthy women, with low and moderate physical activity levels, aged 18 to 35, participated in this study. The inclusion criteria were completion of the participation agreement, female sex, and general good health. Exclusion criteria were metal elements in the body. The study was performed form the beginning of 2013 to the end of 2017 among students and employees of the Medical University of Silesia, as well as families and friends of the mentioned groups. Volunteers fulfilling all of the inclusion and none of the exclusion criteria were included in the study group. The study was conducted in accordance with the Helsinki Declaration. Every participant provided written consent after being informed about the aim, the protocol, and the methods of the study. The research project was approved by the Bioethics Committee of the Medical University of Silesia (KNW/0022/KB1/49/13).

The general characteristics of all study participants are presented in Table 1, and detailed characteristics of the body composition of women with underweight, normal body weight, overweight, and obesity are presented in the Supplementary Material in Tables ESM_1–4.

**Table 1:** General characteristics of all study participants, including BMI classification

Group acc. to. BMI	N		Age [years]	Height [cm]	Weight [kg]	BMI [kg/(m)^2^]
Underweight	37	x ± sd	20.5 ± 2.3	166.6 ± 5.1	48.7 ± 3.3	17.5 ± 0.7
		min; max	19; 30	156; 176	42.9; 56.0	15.9; 18.4
Normal weight	199	x ± sd	21.1 ± 2.7	166.8 ± 5.8	58.5 ± 6.4	21.0 ± 1.8
		min; max	18; 35	153; 180	46.0; 76.7	18.5; 25.0
Overweight	24	x ± sd	23.1 ± 4.6	164.9 ± 5.9	72.3 ± 6.9	26.5 ± 1.3
		min; max	19; 34	155; 178	60.6; 89.4	25.1; 29.6
Obesity	7	x ± sd	22.6 ± 3.1	164.3 ± 2.1	87.2 ± 5.4	32.3 ± 1.8
		min; max	19; 27	161; 168	78.5; 95.0	30.3; 34.9

*N* number of women, *x* mean, *sd* standard deviation, *min* minimum, *max* maximum

BIA was performed on all participants at the beginning of the experiment with an InBodyS10 device (ESM_5). Before measurement of body composition, participants’ heights and weights were measured. Subjects did not eat for at least 4 h before the analysis. Immediately before measurements, participants were asked to empty their bladders and then remained in a supine position for 15 min. After that time, electrodes were placed on the thumbs and middle fingers of the right and left hands as well as on the ankles of the right and left lower limbs, and the analysis were performed (ESM_6).

In this study, the following body composition parameters were measured with an InBodyS10 device: percentage body fat (%), VFA (cm^2^), FFM (kg), SLM (kg), SMM (kg), BCM (kg), bone mineral content (BMC) (kg), TBW (l), intracellular body water (l), ECW (l), and ECW/TBW ratio.

The results were collected in an Excel 2007 spreadsheet, and statistical analysis was performed in STATISTICA 10 software.

The analysis of body composition parameters in underweight, normal body weight, overweight, and obese women according to BMI classification is presented as the percentage of women with low, normal, and high content of each component in particular BMI categories.

## Results

### Analysis of body composition parameters in underweight, normal weight, overweight, and obese women according to BMI classification

**Fig. 1:**
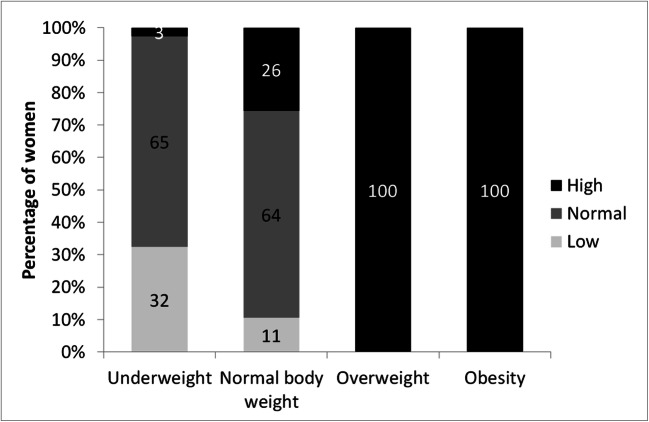
Percentage of women with low, normal and high percentage body fat in underweight, normal weight, overweight and obese women according to BMI classification

In an analysis of the percentage body fat in women, classified into particular categories according to BMI classification, despite being underweight, 3% and 65% of women had high and normal content of fat tissue, respectively (Fig. 1). Approximately 11% of women with normal body weight had a low percentage of body fat, and 26% had a high percentage of body fat. The percentage body fat was high in all overweight and obese women.

All underweight women and 96% of normal weight women had normal VFA. In addition, 43% of obese women and 50% of overweight women, according to BMI classification, had normal VFA.

Approximately 35% of women, despite being underweight according to BMI classification, had normal FFM (Table 2). Among overweight and obese women, 21% and 43%, respectively, were characterized by high FFM.

**Table 2:** Percentage of women with low, normal, and high particular body composition parameters in underweight, normal weight, overweight, and obese women

Body composition parameters	Parameter value	Underweight [%]	Normal weight [%]	Overweight [%]	Obesity [%]
VFA (visceral fat area)	Normal	0	4	50	57
	High	100	96	50	43
FFM (fat-free mass)	High	0	3	21	43
	Normal	35	88	79	57
	Low	65	9	0	0
SLM (soft lean mass)	High	0	2	17	29
	Normal	8	76	79	71
	Low	92	22	4	0
SMM (skeletal muscle mass)	High	0	4	25	43
	Normal	5	71	71	57
	Low	95	25	4	0
BCM (body cell mass)	High	0	4	25	29
	Normal	14	74	71	71
	Low	86	23	4	0
BMC (bone mineral contents)	High	0	23	54	57
	Normal	57	73	46	43
	Low	43	5	0	0
TBW (total body water)	High	0	2	17	29
	Normal	8	76	79	71
	Low	92	22	4	0
ICW (intracellular body water)	High	0	4	25	29
	Normal	14	74	71	71
	Low	86	22	4	0
ECW (extracellular body water)	High	0	1	8	29
	Normal	11	79	92	71
	Low	89	20	0	0
ECW/TBW	High	3	4	4	0
	Normal	97	95	96	100
	Low	0	1	0	0

The analysis of SLM content showed that 17% of overweight women and 29% of women with obesity had high SLM content (Table 2). Most women with excessive BMI had normal SLM. However, 92% of underweight women showed a low content of SLM.

Only 5% of underweight women had normal SMM content. Approximately 25% of women with normal BMI had low muscle mass content, whereas 25% and 43% of overweight and obese women, respectively, had high muscle mass.

A high percentage of underweight women according to the BMI classification were characterized by low BCM. Approximately 23% of women with normal body weight and 4% of overweight women also had a low BCM. A high BCM was found in more than 25% of overweight and 29% of obese women and only 4% of women with normal body weight.

We found a high BMC in 23% of women with normal body weight, 54% of overweight women, and 57% of obese women. Deficiency in BMC occurred in 43% of women with low body weight and 5% of women with normal body weight.

A low volume of TBW was found in 92% of women with low body weight, 22% of women with normal body weight, and 4% of overweight women. A high volume of TBW was found in 2% of women with normal body weight, 17% of overweight women, and 29% of obese women.

Low intracellular water volume was found in 86% of underweight women and 22% of women with normal body weight. Among women with high body weight, a low intracellular water volume occurred only in 4% of individuals.

A low extracellular water volume was found in 89% of underweight women and in 20% of women with normal body weight. The high extracellular volume affected 1% of women with normal body weight, 8% of overweight women, and 29% of obese women.

The ECW/TBW ratio was high in 3% of underweight women, 4% of women with normal body weight, and 4% of overweight women. A low ratio was found in only 1% of women with normal body weight according to BMI classification (Table 2).

## Discussion

Because obesity is associated with excessive BFM, not with the relationship between height and weight, BMI should only serve as an indicator for pre-estimating abnormal weight.

The analysis of individual parameters of body composition in underweight, normal weight, overweight, and obese women according to BMI classification showed the high inaccuracy of this indicator in the diagnosis of body composition disorders. People whose BMI does not correlate with body composition results should be noted. Despite being underweight, 3% of women had a high body fat content. Moreover, 4% of subjects with normal body weight had a high VFA. One person with normal body weight and normal BMI had a VFA of 149.5 cm^2^, which, according to InBodyS10 standards, significantly exceeds the normal value. This result suggests a severe body composition disorder that was undetectable in physical examination. In 1981, Ruderman et al. [9] described patients with metabolically obese normal weight (MONW) syndrome. The diagnostic criteria for MONW syndrome according to Katsuki et al. [10] are the correct BMI with a high VFA percentage. Studies conducted by Romero Corral et al. [11] have shown that MONW syndrome is associated with a high risk of metabolic diseases and an elevated risk of developing cardiovascular diseases. Because the problem affects seemingly healthy and nonobese people, early detection and treatment of the disease are difficult. Bednarek-Tupikowska et al. [12] studied a group of 854 randomly chosen nonobese men and women, 20–40 years of age, selected from three different areas of Poland. All subjects were interviewed and underwent physical examination, anthropometric measurements, and densitometry for total body fat and abdominal fat content. The frequency of MONW is 15.78% in women and 7.83% in men with the criterion for abdominal fat content for women above 30.21% and for men above 28.31%.

Another woman with a high body mass and a BMI indicating obesity had an abdominal fat area of only 72.5 cm^2^. This result indicates a relatively favorable distribution of fat in the body with the dominance of subcutaneous fat within the lower limbs and buttocks. The woman is at lower risk of metabolic diseases such as diabetes, NALFD and cardiovascular diseases, and cancer.

The low diagnostic rate of the BMI was also illustrated by the analysis of other body composition parameters. A deficiency in SMM was found in 25% of people with normal body weight, and 5% also had BMC deficiency. Low muscle mass might have various causes, such as lifestyle (inactivity or inadequate diet) or metabolic and neuroendocrine changes; it is associated with aging, diseases, and obesity (e.g., insulin resistance, inflammation, or oxidative stress) or certain therapies (e.g., chemotherapy) [13].

The results above support the use of body composition analysis in preventive examinations to detect the first irregularities indicating poor health.

The use of BMI has been criticized in publications. Some people have been demonstrated to have a high percentage of body fat but a normal BMI, as also found in this study; moreover, some people identified as obese on the basis of BMI can have low body fat with a high content of lean body mass, e.g., athletes, especially those performing strength training [14].

Because of growing disputes regarding the legitimacy of using BMI in the classification of overweight and obesity, several new indicators have been created in recent years. One of them is the BMI index complemented by the percentage body fat, the so-called BMIfat (BMI adjusted for FM), first proposed by Mialich et al. [15] in 2011. This indicator requires knowledge of the body fat percentage; therefore, an additional measuring device is needed. In the same year, the body adiposity index (BAI) was proposed in the Obesity Journal. In addition to height and weight, the BAI includes hip circumference. Recent studies have demonstrated that the BAI is more reliable than the BMI, which has been used for almost 200 years [16].

In summary, owing to individual differences in body composition, the BMI indicator is not a good tool for illustrating patient health status. It should be replaced by newer anthropometric indicators, and body composition analysis should be performed if possible.

The basic limitation in this research is the method of body composition measurement. The reference method for testing the body composition in humans is dual-energy X-ray absorptiometry (DXA). However, DXA is expensive, time consuming, and difficult to access. BIA is an ideal alternative, because it is relatively inexpensive, fast, easy to use, and safe. Studies comparing DXA and BIA have demonstrated that the BIA method overestimates the content of lean body mass while underestimating BFM [17]. If the DXA method was used, the percentage of people with normal and low BMI but excessive fat content would be even higher.

## Conclusions

In the BMI categories of overweight and obese, only women with excessive BFM are measured with BIA. The BMI category of normal body weight, apart from women with normal body composition, includes people with both deficient and excess body components, e.g., body fat or lean body mass. The BMI category of underweight includes women with different body compositions as well as people with excessive fat content. The BMI index has been shown to be useful as a warning sign of excessive fat content among overweight and obese women, whereas among women with normal body weight and underweight, it may mask various types of body composition irregularities.

## Electronic supplementary material

ESM 1 (DOCX 20 kb)

ESM 2 (DOCX 19 kb)

ESM 3 (DOCX 20 kb)

ESM 4 (DOCX 19 kb)

ESM 5 (JPG 94 kb)

ESM 6 (JPG 230 kb)
