# Supplementary material for: How reliable is BMI? Bioimpedance analysis of body composition in underweight, normal weight, overweight, and obese women
Source: Ir J Med Sci. 2020 Oct 21;190(3):993–8. doi: 10.1007/s11845-020-02403-3 (PMC8302488; doi:10.1007/s11845-020-02403-3)
Supplement: Supplementary file 1 — (DOCX 20 kb) [file 11845_2020_2403_MOESM1_ESM.docx]

ESM_1. Detailed characteristics of body composition in the group of underweight women (N = 37) together with norms; x-average; sd-standard deviation; min-minimum; max-maximum; norm min-lower norm limit in the test group; max norm-upper norm limit in the test group

| Body composition parameters |  | x | sd | min | max |
| --- | --- | --- | --- | --- | --- |
| PBF  Percentage of Body Fat [%] | result | 19.5 | 4.5 | 11.9 | 31.5 |
|  | norm min | - | - | 18.0 | - |
|  | norm max | - | - | - | 28.0 |
| VFA  Visceral Fat Area [cm2] | result | 38.1 | 12.9 | 17.0 | 65.4 |
|  | norm min | - | - | - | - |
|  | norm max | - | - | - | 100.0 |
| FFM Fat Free Mass  [kg] | result | 39.1 | 2.9 | 31.4 | 45.8 |
|  | norm min | 40.2 | 2.7 | 35.3 | 46.2 |
|  | norm max | 50.1 | 3.0 | 44.3 | 56.4 |
| SLM Soft Lean Mass  [kg] | result | 36.8 | 2.7 | 29.3 | 42.9 |
|  | norm min | 39.1 | 2.4 | 34.2 | 43.6 |
|  | norm max | 47.8 | 2.9 | 41.8 | 53.2 |
| SMM Skeletal Muscle Mass [kg] | result | 21.2 | 1.7 | 16.4 | 24.9 |
|  | norm min | 22.8 | 1.5 | 19.7 | 25.6 |
|  | norm max | 27.9 | 1.8 | 24.1 | 31.4 |
| BCM Body Cell Mass  [kg] | result | 25.4 | 1.9 | 20.2 | 29.6 |
|  | norm min | 27.0 | 1.6 | 23.7 | 30.1 |
|  | norm max | 33.0 | 2.0 | 28.9 | 36.8 |
| BMC Bone Mineral Contents [kg] | result | 2.3 | 0.2 | 2.0 | 2.9 |
|  | norm min | 2.3 | 0.1 | 2.0 | 2.6 |
|  | norm max | 2.8 | 0.2 | 2.5 | 3.2 |
| TBW  Total Body Water  [l] | result | 28.7 | 2.1 | 22.8 | 33.4 |
|  | norm min | 30.4 | 1.9 | 26.6 | 33.9 |
|  | norm max | 37.2 | 2.3 | 32.6 | 41.5 |
| ICW Intra-cellular Body Water [l] | result | 17.8 | 1.3 | 14.1 | 20.6 |
|  | norm min | 18.9 | 1.1 | 16.6 | 21.1 |
|  | norm max | 23.0 | 1.4 | 20.2 | 25.7 |
| ECW Extra-cellular Body Water [l] | result | 10.9 | 0.8 | 8.7 | 12.8 |
|  | norm min | 11.6 | 0.7 | 10.2 | 12.9 |
|  | norm max | 14.1 | 0.9 | 12.4 | 15.7 |
| ECW/TBW | result | 0.381 | 0.005 | 0.367 | 0.396 |
|  | norm min | - | - | 0.360 | - |
|  | norm max | - | - | - | 0.390 |
