# Supplementary material for: How reliable is BMI? Bioimpedance analysis of body composition in underweight, normal weight, overweight, and obese women
Source: Ir J Med Sci. 2020 Oct 21;190(3):993–8. doi: 10.1007/s11845-020-02403-3 (PMC8302488; doi:10.1007/s11845-020-02403-3)
Supplement: Supplementary file 2 — (DOCX 19 kb) [file 11845_2020_2403_MOESM2_ESM.docx]

ESM_2. Detailed characteristics of body composition in the group of women with normal body weight (N = 199) together with norms; x-average; sd-standard deviation; min-minimum; max-maximum; norm min-lower norm limit in the test group; max norm-upper norm limit in the test group

| Body composition parameters |  | x | sd | min | max |
| --- | --- | --- | --- | --- | --- |
| PBF  Percentage of Body Fat [%] | result | 24.4 | 5.7 | 10.7 | 38.5 |
|  | norm min | 18.0 | 0.0 | 18.0 | 18.0 |
|  | norm max | 28.0 | 0.0 | 28.0 | 28.0 |
| VFA  Visceral Fat Area [cm2] | result | 55.7 | 21.1 | 6.8 | 149.5 |
|  | norm min | - | - | - | - |
|  | norm max | - | - | - | 100.0 |
| FFM Fat Free Mass  [kg] | result | 44.1 | 4.6 | 32.1 | 56.4 |
|  | norm min | 40.2 | 3.1 | 32.7 | 47.7 |
|  | norm max | 50.2 | 3.5 | 41.8 | 58.3 |
| SLM Soft Lean Mass  [kg] | result | 41.4 | 4.3 | 30.1 | 53.1 |
|  | norm min | 39.2 | 2.7 | 32.9 | 45.5 |
|  | norm max | 47.9 | 3.3 | 40.3 | 55.7 |
| SMM Skeletal Muscle Mass [kg] | result | 24.1 | 2.8 | 17.0 | 31.6 |
|  | norm min | 22.9 | 1.7 | 18.9 | 26.9 |
|  | norm max | 28.0 | 2.1 | 23.1 | 32.9 |
| BCM Body Cell Mass  [kg] | result | 28.7 | 3.1 | 20.8 | 36.9 |
|  | norm min | 27.1 | 1.9 | 22.8 | 31.5 |
|  | norm max | 33.1 | 2.3 | 27.8 | 38.5 |
| BMC Bone Mineral Contents [kg] | result | 2.7 | 0.3 | 2.0 | 3.5 |
|  | norm min | 2.3 | 0.2 | 2.0 | 2.7 |
|  | norm max | 2.8 | 0.2 | 2.4 | 3.3 |
| TBW  Total Body Water  [l] | result | 32.2 | 3.3 | 23.4 | 41.3 |
|  | norm min | 30.5 | 2.1 | 25.6 | 35.5 |
|  | norm max | 37.3 | 2.6 | 31.4 | 43.3 |
| ICW Intra-cellular Body Water [l] | result | 20.0 | 2.1 | 14.5 | 25.8 |
|  | norm min | 18.9 | 1.3 | 15.9 | 22.0 |
|  | norm max | 23.1 | 1.6 | 19.5 | 26.8 |
| ECW Extra-cellular Body Water [l] | result | 12.2 | 1.2 | 8.9 | 15.5 |
|  | norm min | 11.6 | 0.8 | 9.7 | 13.5 |
|  | norm max | 14.2 | 1.0 | 11.9 | 16.5 |
| ECW/TBW | result | 0.379 | 0.007 | 0.351 | 0.395 |
|  | norm min | - | - | 0.360 | - |
|  | norm max | - | - | - | 0.390 |
