# Supplementary material for: How reliable is BMI? Bioimpedance analysis of body composition in underweight, normal weight, overweight, and obese women
Source: Ir J Med Sci. 2020 Oct 21;190(3):993–8. doi: 10.1007/s11845-020-02403-3 (PMC8302488; doi:10.1007/s11845-020-02403-3)
Supplement: Supplementary file 3 — (DOCX 20 kb) [file 11845_2020_2403_MOESM3_ESM.docx]

ESM_3. Detailed characteristics of body composition in the group of overweight women (N = 24) together with norms; x-average; sd-standard deviation; min-minimum; max-maximum; norm min-lower norm limit in the test group; max norm-upper norm limit in the test group

| Body composition parameters |  | x | sd | min | max |
| --- | --- | --- | --- | --- | --- |
| PBF  Percentage of Body Fat [%] | result | 35.3 | 3.5 | 29.1 | 41.5 |
|  | norm min | 18.0 | 0.0 | 18.0 | 18.0 |
|  | norm max | 28.0 | 0.0 | 28.0 | 28.0 |
| VFA  Visceral Fat Area [cm2] | result | 101.3 | 32.9 | 39.1 | 180.9 |
|  | norm min | - | - | - | - |
|  | norm max | - | - | - | 100.0 |
| FFM Fat Free Mass  [kg] | result | 46.8 | 5.1 | 38.6 | 63.3 |
|  | norm min | 39.1 | 3.6 | 33.6 | 47.2 |
|  | norm max | 49.0 | 3.8 | 42.9 | 57.7 |
| SLM Soft Lean Mass  [kg] | result | 44.0 | 4.7 | 36.1 | 59.3 |
|  | norm min | 38.3 | 2.7 | 33.8 | 44.5 |
|  | norm max | 46.8 | 3.4 | 41.3 | 54.5 |
| SMM Skeletal Muscle Mass [kg] | result | 25.7 | 3.1 | 20.4 | 35.6 |
|  | norm min | 22.3 | 1.8 | 19.4 | 26.3 |
|  | norm max | 27.3 | 2.1 | 23.8 | 32.1 |
| BCM Body Cell Mass  [kg] | result | 30.4 | 3.4 | 24.6 | 41.3 |
|  | norm min | 26.5 | 1.9 | 23.4 | 30.8 |
|  | norm max | 32.4 | 2.3 | 28.6 | 37.6 |
| BMC Bone Mineral Contents [kg] | result | 2.8 | 0.3 | 2.3 | 4.0 |
|  | norm min | 2.3 | 0.2 | 2.0 | 2.7 |
|  | norm max | 2.8 | 0.2 | 2.5 | 3.2 |
| TBW  Total Body Water  [l] | result | 34.2 | 3.7 | 28.2 | 46.1 |
|  | norm min | 29.8 | 2.1 | 26.3 | 34.7 |
|  | norm max | 36.4 | 2.6 | 32.1 | 42.5 |
| ICW Intra-cellular Body Water [l] | result | 21.2 | 2.3 | 17.2 | 28.8 |
|  | norm min | 18.5 | 1.3 | 16.3 | 21.5 |
|  | norm max | 22.6 | 1.6 | 19.9 | 26.3 |
| ECW Extra-cellular Body Water [l] | result | 13.0 | 1.3 | 11.0 | 17.3 |
|  | norm min | 11.3 | 0.8 | 10.0 | 13.2 |
|  | norm max | 13.8 | 1.0 | 12.2 | 16.2 |
| ECW/TBW | result | 0.379 | 0.006 | 0.361 | 0.391 |
|  | norm min | - | - | 0.360 | - |
|  | norm max | - | - | - | 0.390 |
