# Supplementary material for: How reliable is BMI? Bioimpedance analysis of body composition in underweight, normal weight, overweight, and obese women
Source: Ir J Med Sci. 2020 Oct 21;190(3):993–8. doi: 10.1007/s11845-020-02403-3 (PMC8302488; doi:10.1007/s11845-020-02403-3)
Supplement: Supplementary file 4 — (DOCX 19 kb) [file 11845_2020_2403_MOESM4_ESM.docx]

ESM_4. Detailed characteristics of body composition in the group of obese women (N = 7) together with norms; x-average; sd-standard deviation; min-minimum; max-maximum; norm min-lower norm limit in the test group; max norm-upper norm limit in the test group

| Body composition parameters |  | x | sd | min | max |
| --- | --- | --- | --- | --- | --- |
| PBF  Percentage of Body Fat [%] | result | 43.4 | 4.2 | 36.7 | 48.4 |
|  | norm min | 18.0 | 0.0 | 18.0 | 18.0 |
|  | norm max | 28.0 | 0.0 | 28.0 | 28.0 |
| VFA  Visceral Fat Area [cm2] | result | 138.8 | 56.8 | 72.5 | 220.5 |
|  | norm min | - | - | - | - |
|  | norm max | - | - | - | 100.0 |
| FFM Fat Free Mass  [kg] | result | 49.3 | 3.2 | 44.8 | 54.4 |
|  | norm min | 38.8 | 1.8 | 36.2 | 40.6 |
|  | norm max | 48.6 | 1.4 | 46.3 | 50.4 |
| SLM Soft Lean Mass  [kg] | result | 46.3 | 2.9 | 42.2 | 51.2 |
|  | norm min | 38.0 | 1.0 | 36.5 | 39.7 |
|  | norm max | 46.4 | 1.2 | 44.5 | 48.5 |
| SMM Skeletal Muscle Mass [kg] | result | 27.4 | 2.0 | 24.8 | 30.7 |
|  | norm min | 22.1 | 0.6 | 21.1 | 23.2 |
|  | norm max | 27.1 | 0.8 | 25.9 | 28.4 |
| BCM Body Cell Mass  [kg] | result | 32.3 | 2.1 | 29.4 | 35.9 |
|  | norm min | 26.3 | 0.7 | 25.2 | 27.5 |
|  | norm max | 32.1 | 0.8 | 30.8 | 33.5 |
| BMC Bone Mineral Contents [kg] | result | 2.9 | 0.3 | 2.6 | 3.4 |
|  | norm min | 2.3 | 0.1 | 2.2 | 2.4 |
|  | norm max | 2.8 | 0.1 | 2.6 | 2.9 |
| TBW  Total Body Water  [l] | result | 36.0 | 2.3 | 32.7 | 39.7 |
|  | norm min | 29.5 | 0.8 | 28.4 | 30.9 |
|  | norm max | 36.1 | 0.9 | 34.7 | 37.7 |
| ICW Intra-cellular Body Water [l] | result | 22.6 | 1.5 | 20.6 | 25.1 |
|  | norm min | 18.3 | 0.5 | 17.6 | 19.2 |
|  | norm max | 22.4 | 0.6 | 21.6 | 23.4 |
| ECW Extra-cellular Body Water [l] | result | 13.4 | 0.8 | 12.1 | 14.6 |
|  | norm min | 11.2 | 0.3 | 10.8 | 11.8 |
|  | norm max | 13.7 | 0.4 | 13.2 | 14.4 |
| ECW/TBW | result | 0.372 | 0.005 | 0.367 | 0.379 |
|  | norm min | - | - | 0.360 | - |
|  | norm max | - | - | - | 0.390 |
