# Supplementary figures and images for: How reliable is BMI? Bioimpedance analysis of body composition in underweight, normal weight, overweight, and obese women
Source: Ir J Med Sci. 2020 Oct 21;190(3):993–8. doi: 10.1007/s11845-020-02403-3 (PMC8302488; doi:10.1007/s11845-020-02403-3)

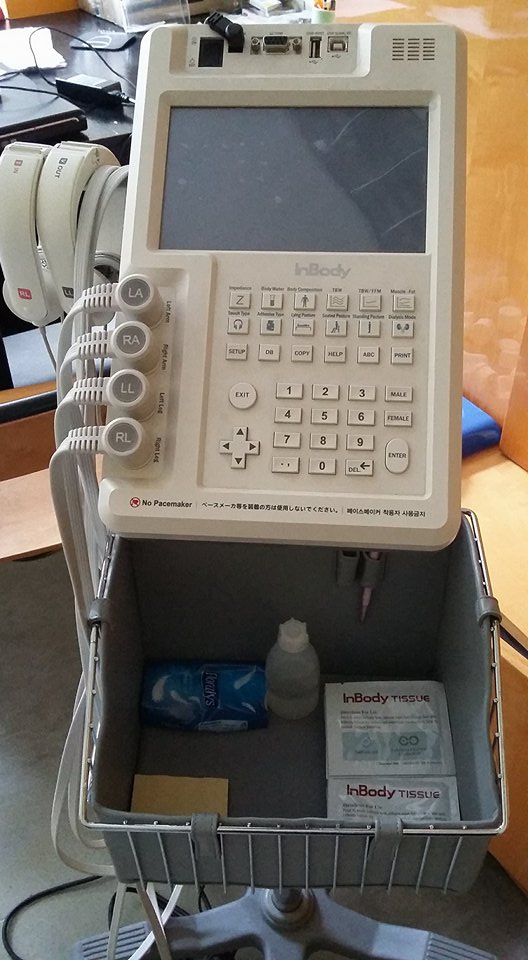

Supplement: Supplementary file 5 — (JPG 94 kb) [file 11845_2020_2403_MOESM5_ESM.jpg]

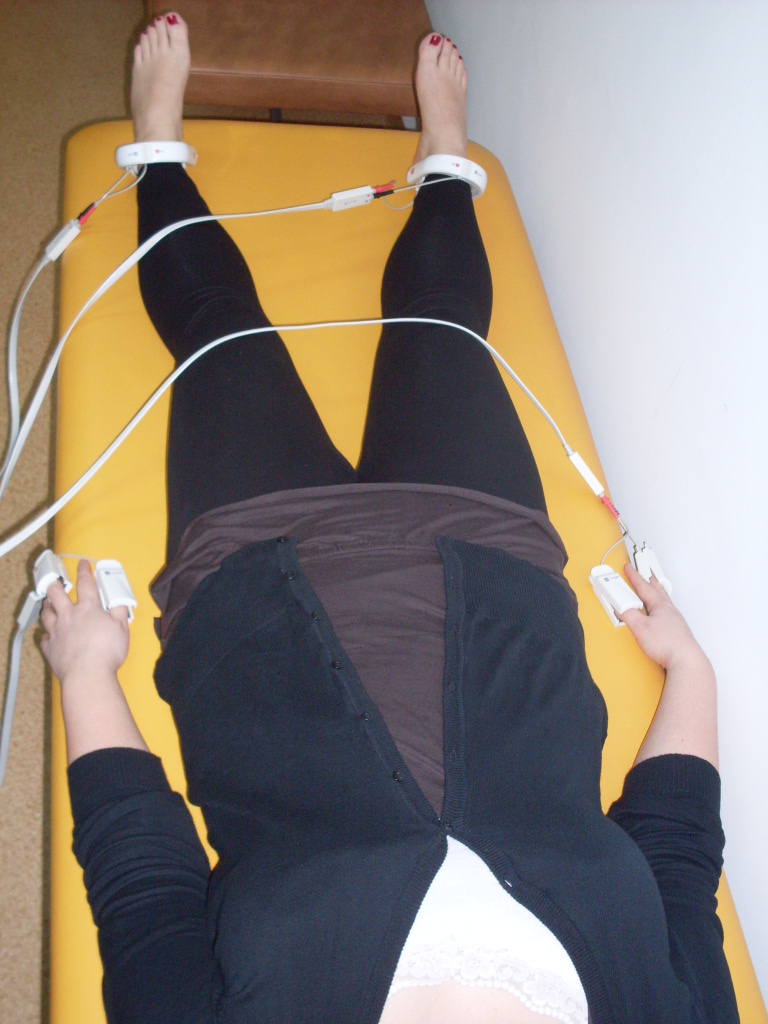

Supplement: Supplementary file 6 — (JPG 230 kb) [file 11845_2020_2403_MOESM6_ESM.jpg]
